# Supplementary material for: The evolutionary processes of bacterial aromatic polyketide ketosynthases
Source: Genome Biol. 2026 Jul 25;27:322. doi: 10.1186/s13059-026-04215-7 (PMC13685256; doi:10.1186/s13059-026-04215-7)
Supplement: Supplementary file 1 [file 13059_2026_4215_MOESM1_ESM.pdf]

ELECTRONIC SUPPLEMENTARY INFORMATION

The evolutionary processes of bacterial aromatic polyketide ketosynthases

Xiaoyu Wang (王小雨)[1#], Qiandi Gao (高千迪)[1#], Liangjun Ge (葛良军)[1], Zhiwei Qin (秦志伟)[1*]

[1]Center for Biological Science and Technology, Advanced Institute of Natural Sciences, Beijing Normal University, Zhuhai, Guangdong, 519087, China.

[#]These authors contributed equally

[*]Correspondence: z.qin@bnu.edu.cn

Table of contents

1. Supplementary Tables

2. Supplementary Figures

1. Supplementary Tables

Table S1. Index and strain information for all 166 T2 PKS KS-CLF pairs in this study. No. 1-166 is assigned to KSs, No. 167-332 is assigned to their corresponding CLFs.

Index	T2PK products name	Building block number	Strain	Accession
0	FABF		*Staphylococcus aureus*	BAD72839.1
1	ksa_A-74528-Hex_13	13	*Streptomyces sp. SANK 61196*	ADG86315.1
2	ksa_Abx_13	13	*Streptomyces sp.*	AUI41025.1
3	ksa_+ABXA-BE-24566B_13	13	*Streptomyces sp.*	KU243130.1
4	ksa_Accramycin_13	13	*Streptomyces sp.*	QKO28696.1
5	ksa_Aclacinomycin'-Pr_10	10	*Streptomyces galilaeus*	AAF70106.1
6	ksa_Aclacinomycin-Pr_10	10	*Streptomyces galilaeus*	BAB72045.1
7	ksa_Actinorhodin_8	8	*Streptomyces coelicolor A3(2)*	CAC44200.1
8	ksa_Allocyclinone_10	10	*Actinoallomurus sp. ID145698*	AMX23327.1
9	ksa_Alnumycin-Bu_8	8	*Streptomyces sp. CM020*	ACI88861.1
10	ksa_Anthrabenzoxocinone_13	13	*Actinobacteria bacterium*	AUO15560.1
11	ksa_AQ-256_8	8	*Photorhabdus laumondii subsp. laumondii TTO1*	CAE16563.1
12	ksa_Aranciamycin_10	10	*Streptomyces echinatus*	ABL09959.1
13	ksa_Arenimycin'_12	12	*uncultured bacterium BAC-AB1442/1414/561*	AIW63010.1
14	ksa_Arenimycin_12	12	*Salinispora arenicola*	WP_029025958.1
15	ksa_Arimetamycin_10	10	*uncultured bacterium*	AHA81977.1
16	ksa_Arixanthomycin_13	13	*uncultured bacterium*	AHX24701.1
17	ksa_Auricin_10	10	*Streptomyces lavendulae subsp. lavendulae*	AAX57191.1

18	ksa_Azicemicin-aziridine_10	10	*Kibdelosporangium sp. MJ126-NF4*	ADB02843.1
19	ksa_Baikalomycin_10	10	*Streptomyces sp. IB201691-2A2*	WP_143644093.1
20	ksa_BE-7585A_10	10	*Amycolatopsis orientalis subsp. vinearia*	ADI71443.1
21	ksa_Benastatin-Hex_12	12	*Streptomyces sp. A2991200*	CAM58798.1
22	ksa_Bipentaromycin_10	10	*Streptomyces sp. NRRL F-6131*	WP_030301373.1
23	ksa_Brasiliquinone-Pr_10	10	*Nocardia brasiliensis*	WP_146154178.1
24	ksa_Calixanthomycin_12	12	*uncultured bacterium*	AJD20010.1
25	ksa_Cervimycin-malonoamyl_9	9	*Streptomyces sp. CS113*	OWA01596.1
26	ksa_Chartreusin'_10	10	*Streptomyces chartreusis*	AXS67802.1
27	ksa_Chartreusin_10	10	*Streptomyces chartreusis*	CAH10161.1
28	ksa_Chattamycin_10	10	*Streptomyces lydicus*	WP_046929079.1
29	ksa_Chelocardin_10	10	*Amycolatopsis sulphurea*	AHD25926.1
30	ksa_Chlortetracycline_10	10	*Kitasatospora aureofaciens*	AEI98666.1
31	ksa_Chromomycin_10	10	*Streptomyces griseus subsp. griseus*	CAE17527.1
32	ksa_Chrysomycin-Pr_10	10	*Streptomyces albaduncus*	CBH32088.1
33	ksa_Cinerubin-Pr_10	10	*Streptomyces sp. SPB074*	EDY42532.1
34	ksa_Clostrubin'_X	10	*Clostridium puniceum*	WP_077846908.1
35	ksa_Clostrubin_X	10	*Clostridium beijerinckii*	WP_077837156.1
36	ksa_Collinone_13	13	*Streptomyces collinus*	AAG26879.1
37	ksa_CosmomycinC-Pr_10	10	*Streptomyces sp. CNT302*	WP_017947381.1
38	ksa_Cosmomycin-Pr_10	10	*Streptomyces olindensis*	ABC00726.1
39	ksa_Cur_12	12	*Streptomyces cyaneus*	CAA44380.1
40	ksa_Cysgriseusin_10	10	*Streptomyces cyaneofuscatus*	WP_030562167.1

41	ksa_Cytorhodin-Pr_10	10	*Streptomyces sp. SCSIO 1666*	ATJ00755.1
42	ksa_Dactylocycline_10	10	*Dactylosporangium sp. SC14051*	AFU65894.1
43	ksa_Daunorubicin-Pr_10	10	*Streptomyces sp.*	AAA87618.1
44	ksa_Dendrubin_9	9	*Dendrosporobacter quercicolus*	WP_092069528.1
45	ksa_Doxorubicin'-Pr_10	10	*Streptomyces peucetius*	WP_100109102.1
46	ksa_Doxorubicin-Pr_10	10	*Streptomyces peucetius*	AAA65206.1
47	ksa_Dutomycin_10	10	*Streptomyces minoensis*	AKD43514.1
48	ksa_Elloramycin_10	10	*Streptomyces olivaceus*	CAP12600.1
49	ksa_Enduracyclinone_13	13	*Nonomuraea sp.*	AYP71359.1
50	ksa_Enterocin-Bn_8	8	*Streptomyces maritimus*	AAF81728.1
51	ksa_Erdacin_8	8	*uncultured soil bacterium V167*	ACX83617.1
52	ksa_Fasamycin_13	13	*N/A*	AEM44268.1
53	ksa_FD-594-Bu_13	13	*Streptomyces sp. TA-0256*	BAJ52681.1
54	ksa_Fluostatin_10	10	*uncultured bacterium BAC AB649/1850*	AEE65468.1
55	ksa_FluostatinM_10	10	*Micromonospora rosaria*	ALJ99855.1
56	ksa_FluostatinMQ_10	10	*Streptomyces albus*	WP_040253443.1
57	ksa_Fogacin_8	8	*Actinoplanes missouriensis 431*	BAL90284.1
58	ksa_FogacinC-HCS_8	8	*Actinoplanes missouriensis 431*	BAL90263.1
59	ksa_Formicamycin_13	13	*Streptomyces sp. KY5*	AQP25565.1
60	ksa_Frankiamicin_12	12	*Frankia sp. EAN1pec*	ABW11825.1
61	ksa_FredericamycinC_13	13	*Streptomyces albus subsp. chlorinus*	NSC21571.1
62	ksa_Fredericamycin-Hex_13unknown	13	*Streptomyces griseus*	AAQ08916.1
63	ksa_Frenolicin-Bu_8unknown	8	*Streptomyces roseofulvus*	AAC18107.1
64	ksa_Frigocyclinone_10unknown	10	*Streptomyces griseus*	QDG00826.1

65	ksa_Gaudimycin_10	10	*Streptomyces sp. PGA64*	AAK57525.1
66	ksa_Gilvocarcin-Pr_10	10	*Streptomyces griseoflavus*	AAP69573.1
67	ksa_Granaticin''_8unknown	8	*Streptomyces exfoliatus*	WP_137988251.1
68	ksa_Granaticin'_8unknown	8	*Streptomyces violaceoruber*	CAA09653.1
69	ksa_Granaticin_8unknown	8	*Streptomyces vietnamensis*	ADO32786.1
70	ksa_Grincamycin_10	10	*Streptomyces lusitanus*	AGO50610.1
71	ksa_Griseorhodin_13	13	*Streptomyces sp. JP95*	AAM33653.1
72	ksa_Griseusin_10unknown	10	*Streptomyces griseus*	CAA54858.1
73	ksa_Hatomarubigin_10unknown	10	*Streptomyces sp. 2238-SVT4*	BAJ07842.1
74	ksa_Hedamycin-Hex_10	10	*Streptomyces griseoruber*	AAP85362.1
75	ksa_Heliquinomycin_13unknown	13	*Streptomyces piniterrae*	WP_136741436.
76	ksa_Hexaricin_13	13	*Streptosporangium sp. FXJ7.131*	AMK51281.1
77	ksa_Hiroshidine_8	8	*Streptomyces hiroshimensis*	QBK46637.1
78	ksa_Huanglongmycin_9unknown	9	*Streptomyces sp. CB09001*	AXL88816.1
79	ksa_Hyaluromycin_13unknown	13	*Streptomyces hyaluromycini*	WP_089099014.
80	ksa_Hydroxyfujianmycin_10unknown	10	*Streptomyces alni*	WP_093715797.
81	ksa_IFQs_8	8	*Streptomyces sp. CB01883*	APR73637.1
82	ksa_Isatropolone-Bu_8unknown	8	*Streptomyces sp.*	ARG41911.1
83	ksa_Isoindolinomycin-Gly_8unknown	8	*Streptomyces sp. SoC090715LN-16*	BBE36466.1
84	ksa_Jadomycin_10unknown	10	*Streptomyces venezuelae ATCC 10712*	AAB36562.1
85	ksa_JBIR-76_8	8	*Streptomyces sp. RI-77*	BAU98041.1
86	ksa_Julichrome'_9unknown	9	*Streptomyces sampsonii*	QNL10613.1
87	ksa_Julichrome_9unknown	9	*Streptomyces afghaniensis*	WP_020275095.
88	ksa_Keyicin_10unknown	10	*Micromonospora sp. WMMB235*	OHX01516.1

89	ksa_Kiamycin_9unknown	9	*Streptomyces sp. W007*	EHM27505.1
90	ksa_Kinamycin_10unknown	10	*Streptomyces murayamaensis*	AAO65346.1
91	ksa_kinanthraquinone_9unknown	9	*Streptomyces sp. SN-593*	WP_202233564.
92	ksa_Komodoquinone'_10unknown	10	*Streptomyces erythrochromogenes*	WP_031147015.
93	ksa_Komodoquinone_10unknown	10	*Streptomyces sp. NRRL S-378*	WP_030957354.1
94	ksa_Kosinostatin_10	10	*Micromonospora sp. TP-A0468*	AFJ52674.1
95	ksa_Lactonamycin-Gly_10	10	*Streptomyces rishiriensis*	ABX71114.1
96	ksa_LactonamycinZ-Gly_10	10	*Streptomyces sanglieri*	ABX71148.1
97	ksa_Landomycin_10unknown	10	*Streptomyces cyanogenus*	AAD13536.1
98	ksa_LandomycinE'_10unknown	10	*uncultured bacterium*	AEM44218.1
99	ksa_LandomycinE_10unknown	10	*Streptomyces globisporus*	AID46990.1
100	ksa_LL-D49194_1-Ile_10	10	*Streptomyces vinaceusdrappus*	QDQ37910.1
101	ksa_Lomaiviticin''-Pr_10unknown	10	*Salinispora pacifica*	AHZ61892.1
102	ksa_Lomaiviticin'-Pr_10unknown	10	*Salinispora pacifica*	AHF72794.1
103	ksa_Lomaiviticin-Pr_10unknown	10	*Salinispora tropica CNB-440*	ABP54674.1
104	ksa_Lugdunomycin_10unknown	10	*Streptomyces sp. QL37*	PPQ57489.1
105	ksa_Lysolipin_12	12	*Streptomyces tendae*	CAM34345.1
106	ksa_Maduralactomycin_10unknown	10	*Actinomadura rubteroloni*	WP_103564581.1
107	ksa_Mayamycin_10	10	*Streptomyces sp.*	AVO00815.1
108	ksa_Medermycin_8	8	*Streptomyces sp. AM-7161*	BAC79044.1
109	ksa_Mensacaricin_10unknown	10	*Streptomyces bottropensis*	AHL46686.1
110	ksa_Metamycin-iPr_89unknown	89	*Rothia dentocariosa*	VEJ30651.1
111	ksa_Metathramycin_10unknown	10	*uncultured bacterium*	QVQ68799.1
112	ksa_Methyltetrangomycin_10unknown	10	*Streptomyces sp. Go-475*	WP_114256228.1

113	ksa_Mithramycin_10unknown	10	*Streptomyces argillaceus*	CAA61989.1
114	ksa_Murayaquinone_10unknown	10	*Streptomyces griseoruber*	AQW35058.1
115	ksa_Naphthocyclinone_8unknown	8	*Streptomyces arenae*	AAD20267.1
116	ksa_Nenestatin-Pr_10unknown	10	*Micromonospora echinospora*	ARD70901.1
117	ksa_Nivetetracyclate-Pr_10unknown	10	*Streptomyces sp. Ls2151*	AGZ78377.1
118	ksa_Nocardiopsistin-iPr_10unknown	10	*Nocardiopsis sp.*	QOP59270.1
119	ksa_Nogalamycin_10unknown	10	*Streptomyces nogalater*	CAA12017.1
120	ksa_Oviedomycin'_10	10	*Streptomyces antibioticus*	ARK36155.1
121	ksa_Oviedomycin_10	10	*Streptomyces antibioticus*	CAG14965.1
122	ksa_Oxytetracycline'_10unknown	10	*Streptomyces rimosus subsp. rimosus ATCC 10970*	ELQ83269.1
123	ksa_Oxytetracycline_10unknown	10	*Streptomyces rimosus*	AAZ78325.1
124	ksa_Paramagnetoquinone_Xunknown	10	*Actinoallomurus sp.*	ASA49564.1
125	ksa_PD116740_10unknown	10	*Streptomyces sp. WP 4669*	AAO65362.1
126	ksa_Persiamycin_10unknown	10	*Streptomonospora sp. PA3*	WP_156000395.1
127	ksa_Piloquinone-Leu_9	9	*Streptomyces sp. FXJ8.102*	QFS19044.1
128	ksa_Polyketomycin_10	10	*Streptomyces diastatochromogenes*	ACN64834.1
129	ksa_Pradimicin_12unknown	12	*Actinomadura hibisca*	ABM21747.1
130	ksa_Pyxidicycline_9	9	*Pyxidicoccus fallax*	AXM42927.1
131	ksa_Qinimycin_8unknown	8	*Streptomyces roseifaciens*	WP_058047374.1
132	ksa_R1128-Leu_8unknown	8	*Streptomyces sp. R1128*	AAG30189.1
133	ksa_Ravidomycin-Pr_10unknown	10	*Streptomyces ravidus*	CBH32809.1
134	ksa_Resistomycin_X	10	*Streptomyces resistomycificus*	CAE51174.1
135	ksa_Rhodomycin-Pr_10unknown	10	*Streptomyces purpurascens*	WP_189725834.1

136	ksa_Rishirilide'-Leu_9unknown	9	*Streptomyces olivaceus*	AWM72904.1
137	ksa_Rishirilide-Leu_9unknown	9	*Streptomyces bottropensis*	AHL46710.1
138	ksa_Rubrolone-Bu_8unknown	8	*Streptomyces sp. KIB-H033*	AOZ61212.1
139	ksa_Rubromycin_13	13	*Streptomyces collinus*	WP_073806072.1
140	ksa_Rubromycin'_13unknown	13	*unclassified Streptomyces*	AAG03067.1
141	ksa_Saprolmycin_10unknown	10	*Streptomyces sp. TK08046*	BAV16999.1
142	ksa_SaquayamycinA_10	10	*Streptomyces sp.*	ARO44655.1
143	ksa_SaquayamycinZ_10	10	*Micromonospora sp. Tu 6368*	ACP19353.1
144	ksa_Sch_12unknown	12	*Streptomyces halstedii*	AAA02833.1
145	ksa_Sch47554_10unknown	10	*Streptomyces sp. SCC 2136*	CAH10117.1
146	ksa_Setomimycin_9unknown	9	*Streptomyces aurantiacus*	WP_037658548.1
147	ksa_SF2575_10	10	*Streptomyces sp. SF2575*	ADE34518.1
148	ksa_Simocyclinone'_10unknown	10	*Streptomyces sp. NRRL B-24484*	WP_052390556.1
149	ksa_Simocyclinone_10unknown	10	*Streptomyces antibioticus*	AAK06784.1
150	ksa_Steffimycin_10unknown	10	*Streptomyces steffisburgensis*	CAJ42320.1
151	ksa_Streptoketide-Pr_10unknown	10	*Streptomyces sp. Tue6314*	QED90637.1
152	ksa_Tetarimycin_10	10	*uncultured bacterium*	AFY23044.1
153	ksa_Tetracenomycin_10	10	*Streptomyces glaucescens*	AAA67515.1
154	ksa_Thioangucycline_10	10	*Streptomyces sp. CB00072*	WP_073867016.1
155	ksa_TLN-05220-Ile_13	13	*Micromonospora echinospora subsp. challisensis*	ADB23391.1
156	ksa_Trioxacarcin-Ile_10	10	*Streptomyces bottropensis*	AKT74262.1
157	ksa_Turbinmicin_13	13	*Micromonospora sp. WMMC415*	WP_155333336.1
158	ksa_Urdamycin_10	10	*Streptomyces fradiae*	CAA60569.1
159	ksa_UT-X26F129_10	10	*Streptomyces aureus*	AYU66233.1

160	ksa_Warkmycin_10	10	*Streptomyces sp. CS057*	OWA25245.1
161	ksa_Wexrubicin_X	10	*Eubacteriales*	WP_025578803.1
162	ksa_WhiE_12	12	*Streptomyces coelicolor*	CAA39408.1
163	ksa_WS79089D_13	13	*Streptosporangium roseum DSM 43021*	WP_2890796.1
164	ksa_X26_10	10	*uncultured bacterium*	AEM44309.1
165	ksa_Xantholipin_13	13	*Streptomyces flavogriseus*	ADE22315.1
166	ksa_ZHMpentangular_13	13	*Streptomyces viridochromogenes*	WP_003993028.1
167	ksb_A-74528-Hex_13	13	*Streptomyces sp. SANK 61196*	ADG86316.1
168	ksb_Abx_13	13	*Streptomyces sp.*	AUI41026.1
169	ksb_+ABXA-BE-24566B_13	13	*Streptomyces sp.*	KU243130.1
170	ksb_Accramycin_13	13	*Streptomyces sp.*	QKO28695.1
171	ksb_Aclacinomycin'-Pr_10	10	*Streptomyces galilaeus*	AAF70107.1
172	ksb_Aclacinomycin-Pr_10	10	*Streptomyces galilaeus*	BAB72046.1
173	ksb_Actinorhodin_8	8	*Streptomyces coelicolor A3(2)*	CAC44201.1
174	ksb_Allocyclinone_10	10	*Actinoallomurus sp. ID145698*	AMX23328.1
175	ksb_Alnumycin-Bu_8	8	*Streptomyces sp. CM020*	ACI88862.1
176	ksb_Anthrabenzoxocinone_13	13	*Actinobacteria bacterium*	AUO15561.1
177	ksb_AQ-256_8	8	*Photorhabdus laumondii subsp. laumondii TTO1*	CAE16562.1
178	ksb_Aranciamycin_10	10	*Streptomyces echinatus*	ABL09958.1
179	ksb_Arenimycin'_12	12	*uncultured bacterium BAC-AB1442/1414/561*	AIW63011.1
180	ksb_Arenimycin_12	12	*Salinispora arenicola*	WP_029025959.1
181	ksb_Arimetamycin_10	10	*uncultured bacterium*	AHA81978.1
182	ksb_Arixanthomycin_13	13	*uncultured bacterium*	AHX24702.1

183	ksb_Auricin_10	10	*Streptomyces lavendulae subsp. lavendulae*	AAX57192.2
184	ksb_Azicemicin-aziridine_10	10	*Kibdelosporangium sp. MJ126-NF4*	ADB02844.1
185	ksb_Baikalomycin_10	10	*Streptomyces sp. IB201691-2A2*	WP_143644094.1
186	ksb_BE-7585A_10	10	*Amycolatopsis orientalis subsp. vinearia*	ADI71444.1
187	ksb_Benastatin-Hex_12	12	*Streptomyces sp. A2991200*	CAM58799.1
188	ksb_Bipentaromycin_10	10	*Streptomyces sp. NRRL F-6131*	WP_030301370.1
189	ksb_Brasiliquinone-Pr_10	10	*Nocardia brasiliensis*	WP_042264335.1
190	ksb_Calixanthomycin_12	12	*uncultured bacterium*	AJD20009.1
191	ksb_Cervimycin-malonoamyl_9	9	*Streptomyces sp. CS113*	OWA01597.1
192	ksb_Chartreusin'_10	10	*Streptomyces chartreusis*	AXS67803.1
193	ksb_Chartreusin_10	10	*Streptomyces chartreusis*	CAH10162.1
194	ksb_Chattamycin_10	10	*Streptomyces lydicus*	WP_046929080.1
195	ksb_Chelocardin_10	10	*Amycolatopsis sulphurea*	AHD25927.1
196	ksb_Chlortetracycline_10	10	*Kitasatospora aureofaciens*	AEI98665.1
197	ksb_Chromomycin_10	10	*Streptomyces griseus subsp. griseus*	CAE17526.1
198	ksb_Chrysomycin-Pr_10	10	*Streptomyces albaduncus*	CBH32089.1
199	ksb_Cinerubin-Pr_10	10	*Streptomyces sp. SPB074*	EDY42531.1
200	ksb_Clostrubin'_X	10	*Clostridium puniceum*	WP_077846909.1
201	ksb_Clostrubin_X	10	*Clostridium beijerinckii*	WP_077837155.1
202	ksb_Collinone_13	13	*Streptomyces collinus*	AAG26880.1
203	ksb_CosmomycinC-Pr_10	10	*Streptomyces sp. CNT302*	WP_018891747.1
204	ksb_Cosmomycin-Pr_10	10	*Streptomyces olindensis*	ABC00728.1
205	ksb_Cur_12	12	*Streptomyces cyaneus*	CAA44381.1

206	ksb_Cysgriseusin_10	10	*Streptomyces cyaneofuscatus*	WP_030562169.1
207	ksb_Cytorhodin-Pr_10	10	*Streptomyces sp. SCSIO 1666*	ATJ00754.1
208	ksb_Dactylocycline_10	10	*Dactylosporangium sp. SC14051*	AFU65895.1
209	ksb_Daunorubicin-Pr_10	10	*Streptomyces sp.*	AAA87619.1
210	ksb_Dendrubin_9	9	*Dendrosporobacter quercicolus*	WP_092069525.1
211	ksb_Doxorubicin'-Pr_10	10	*Streptomyces peucetius*	WP_100109101.1
212	ksb_Doxorubicin-Pr_10	10	*Streptomyces peucetius*	AAA65207.1
213	ksb_Dutomycin_10	10	*Streptomyces minoensis*	AKD43513.1
214	ksb_Elloramycin_10	10	*Streptomyces olivaceus*	CAP12601.1
215	ksb_Enduracyclinone_13	13	*Nonomuraea sp.*	AYP71360.1
216	ksb_Enterocin-Bn_8	8	*Streptomyces maritimus*	AAF81729.1
217	ksb_Erdacin_8	8	*uncultured soil bacterium V167*	ACX83618.1
218	ksb_Fasamycin_13	13	*N/A*	AEM44267.1
219	ksb_FD-594-Bu_13	13	*Streptomyces sp. TA-0256*	BAJ52682.1
220	ksb_Fluostatin_10	10	*uncultured bacterium BAC AB649/1850*	AEE65467.1
221	ksb_FluostatinM_10	10	*Micromonospora rosaria*	ALJ99854.1
222	ksb_FluostatinMQ_10	10	*Streptomyces albus*	WP_040253445.1
223	ksb_Fogacin_8	8	*Actinoplanes missouriensis 431*	BAL90285.1
224	ksb_FogacinC-HCS_8	8	*Actinoplanes missouriensis 431*	BAL90262.1
225	ksb_Formicamycin_13	13	*Streptomyces sp. KY5*	AQP25564.1
226	ksb_Frankiamicin_12	12	*Frankia sp. EAN1pec*	ABW11826.1
227	ksb_FredericamycinC_13	13	*Streptomyces albus subsp. chlorinus*	NSC21570.1
228	ksb_Fredericamycin-Hex_13	13	*Streptomyces griseus*	AAQ08917.1
229	ksb_Frenolicin-Bu_8	8	*Streptomyces roseofulvus*	AAC18108.1

230	ksb_Frigocyclinone_10	10	*Streptomyces griseus*	QDG00825.1
231	ksb_Gaudimycin_10	10	*Streptomyces sp. PGA64*	AAK57526.1
232	ksb_Gilvocarcin-Pr_10	10	*Streptomyces griseoflavus*	AAP69574.1
233	ksb_Granaticin''_8	8	*Streptomyces exfoliatus*	WP_137988252.1
234	ksb_Granaticin'_8	8	*Streptomyces violaceoruber*	CAA09654.1
235	ksb_Granaticin_8	8	*Streptomyces vietnamensis*	ADO32787.1
236	ksb_Grincamycin_10	10	*Streptomyces lusitanus*	AGO50611.1
237	ksb_Griseorhodin_13	13	*Streptomyces sp. JP95*	AAM33654.1
238	ksb_Griseusin_10	10	*Streptomyces griseus*	CAA54859.1
239	ksb_Hatomarubigin_10	10	*Streptomyces sp. 2238-SVT4*	BAJ07844.1
240	ksb_Hedamycin-Hex_10	10	*Streptomyces griseoruber*	AAP85361.1
241	ksb_Heliquinomycin_13	13	*Streptomyces piniterrae*	WP_136741437.1
242	ksb_Hexaricin_13	13	*Streptosporangium sp. FXJ7.131*	AMK51280.1
243	ksb_Hiroshidine_8	8	*Streptomyces hiroshimensis*	QBK46636.1
244	ksb_Huanglongmycin_9	9	*Streptomyces sp. CB09001*	AXL88815.1
245	ksb_Hyaluromycin_13	13	*Streptomyces hyaluromycini*	WP_089099013.1
246	ksb_Hydroxyfujianmycin_10	10	*Streptomyces alni*	WP_093715798.1
247	ksb_IFQs_8	8	*Streptomyces sp. CB01883*	APR73638.1
248	ksb_Isatropolone-Bu_8	8	*Streptomyces sp.*	ARG41912.1
249	ksb_Isoindolinomycin-Gly_8	8	*Streptomyces sp. SoC090715LN-16*	BBE36465.1
250	ksb_Jadomycin_10	10	*Streptomyces venezuelae ATCC 10712*	AAB36563.1
251	ksb_JBIR-76_8	8	*Streptomyces sp. RI-77*	BAU98042.1
252	ksb_Julichrome'_9	9	*Streptomyces sampsonii*	QNL10614.1
253	ksb_Julichrome_9	9	*Streptomyces afghaniensis*	WP_037671486.1

254	ksb_Keyicin_10	10	*Micromonospora sp. WMMB235*	OHX01517.1
255	ksb_Kiamycin_9	9	*Streptomyces sp. W007*	EHM27506.1
256	ksb_Kinamycin_10	10	*Streptomyces murayamaensis*	AAO65347.1
257	ksb_kinanthraquinone_9	9	*Streptomyces sp. SN-593*	WP_202233565.1
258	ksb_Komodoquinone'_10	10	*Streptomyces erythrochromogenes*	WP_031147017.1
259	ksb_Komodoquinone_10	10	*Streptomyces sp. NRRL S-378*	WP_030957356.1
260	ksb_Kosinostatin_10	10	*Micromonospora sp. TP-A0468*	AFJ52675.1
261	ksb_Lactonamycin-Gly_10	10	*Streptomyces rishiriensis*	ABX71115.1
262	ksb_LactonamycinZ-Gly_10	10	*Streptomyces sanglieri*	ABX71149.1
263	ksb_Landomycin_10	10	*Streptomyces cyanogenus*	AAD13537.1
264	ksb_LandomycinE'_10	10	*uncultured bacterium*	AEM44219.1
265	ksb_LandomycinE_10	10	*Streptomyces globisporus*	AID46991.1
266	ksb_LL-D49194_1-Ile_10	10	*Streptomyces vinaceusdrappus*	QDQ37911.1
267	ksb_Lomaiviticin''-Pr_10	10	*Salinispora pacifica*	AHZ61893.1
268	ksb_Lomaiviticin'-Pr_10	10	*Salinispora pacifica*	AHF72795.1
269	ksb_Lomaiviticin-Pr_10	10	*Salinispora tropica CNB-440*	ABP54675.1
270	ksb_Lugdunomycin_10	10	*Streptomyces sp. QL37*	PPQ57490.1
271	ksb_Lysolipin_12	12	*Streptomyces tendae*	CAM34344.1
272	ksb_Maduralactomycin_10	10	*Actinomadura rubteroloni*	WP_103562971.1
273	ksb_Mayamycin_10	10	*Streptomyces sp.*	AVO00814.1
274	ksb_Medermycin_8	8	*Streptomyces sp. AM-7161*	BAC79045.1
275	ksb_Mensacaricin_10	10	*Streptomyces bottropensis*	AHL46685.1
276	ksb_Metamycin-iPr_8_9	8/9	*Rothia dentocariosa*	VEJ30650.1
277	ksb_Metathramycin_10	10	*uncultured bacterium*	QVQ68798.1

278	ksb_Methyltetrangomycin_10	10	*Streptomyces sp. Go-475*	WP_114256227.1
279	ksb_Mithramycin_10	10	*Streptomyces argillaceus*	CAA61990.1
280	ksb_Murayaquinone_10	10	*Streptomyces griseoruber*	AQW35059.1
281	ksb_Naphthocyclinone_8	8	*Streptomyces arenae*	AAD20268.1
282	ksb_Nenestatin-Pr_10	10	*Micromonospora echinospora*	ARD70902.1
283	ksb_Nivetetracyclate-Pr_10	10	*Streptomyces sp. Ls2151*	AGZ78375.1
284	ksb_Nocardiopsistin-iPr_10	10	*Nocardiopsis sp.*	QOP59271.1
285	ksb_Nogalamycin_10	10	*Streptomyces nogalater*	CAA12018.1
286	ksb_Oviedomycin'_10	10	*Streptomyces antibioticus*	ARK36156.1
287	ksb_Oviedomycin_10	10	*Streptomyces antibioticus*	CAG14966.1
288	ksb_Oxytetracycline'_10	10	*Streptomyces rimosus subsp. rimosus ATCC 10970*	ELQ83293.1
289	ksb_Oxytetracycline_10	10	*Streptomyces rimosus*	AAZ78326.1
290	ksb_Paramagnetoquinone_X	10	*Actinoallomurus sp.*	ASA49565.1
291	ksb_PD116740_10	10	*Streptomyces sp. WP 4669*	AAO65363.1
292	ksb_Persiamycin_10	10	*Streptomonospora sp. PA3*	WP_156000393.1
293	ksb_Piloquinone-Leu_9	9	*Streptomyces sp. FXJ8.102*	QFS19045.1
294	ksb_Polyketomycin_10	10	*Streptomyces diastatochromogenes*	ACN64835.1
295	ksb_Pradimicin_12	12	*Actinomadura hibisca*	ABM21748.1
296	ksb_Pyxidicycline_9	9	*Pyxidicoccus fallax*	AXM42928.1
297	ksb_Qinimycin_8	8	*Streptomyces roseifaciens*	WP_058047373.1
298	ksb_R1128-Leu_8	8	*Streptomyces sp. R1128*	AAG30188.1
299	ksb_Ravidomycin-Pr_10	10	*Streptomyces ravidus*	CBH32808.1
300	ksb_Resistomycin_X	10	*Streptomyces resistomycificus*	CAE51175.1

301	ksb_Rhodomycin-Pr_10	10	*Streptomyces purpurascens*	WP_189725835.1
302	ksb_Rishirilide'-Leu_9	9	*Streptomyces olivaceus*	AWM72903.1
303	ksb_Rishirilide-Leu_9	9	*Streptomyces bottropensis*	AHL46709.1
304	ksb_Rubrolone-Bu_8	8	*Streptomyces sp. KIB-H033*	AOZ61213.1
305	ksb_Rubromycin'_13	13	*Streptomyces collinus*	WP_073806071.1
306	ksb_Rubromycin_13	13	*unclassified Streptomyces*	AAG03068.1
307	ksb_Saprolmycin_10	10	*Streptomyces sp. TK08046*	BAV17000.1
308	ksb_SaquayamycinA_10	10	*Streptomyces sp.*	ARO44656.1
309	ksb_SaquayamycinZ_10	10	*Micromonospora sp. Tu 6368*	ACP19354.1
310	ksb_Sch_12	12	*Streptomyces halstedii*	AAA02834.1
311	ksb_Sch47554_10	10	*Streptomyces sp. SCC 2136*	CAH10116.1
312	ksb_Setomimycin_9	9	*Streptomyces aurantiacus*	WP_037658550.1
313	ksb_SF2575_10	10	*Streptomyces sp. SF2575*	ADE34519.1
314	ksb_Simocyclinone'_10	10	*Streptomyces sp. NRRL B-24484*	WP_030269265.1
315	ksb_Simocyclinone_10	10	*Streptomyces antibioticus*	AAK06785.1
316	ksb_Steffimycin_10	10	*Streptomyces steffisburgensis*	CAJ42319.1
317	ksb_Streptoketide-Pr_10	10	*Streptomyces sp. Tue6314*	QED90612.1
318	ksb_Tetarimycin_10	10	*uncultured bacterium*	AFY23043.1
319	ksb_Tetracenomycin_10	10	*Streptomyces glaucescens*	AAA67516.1
320	ksb_Thioangucycline_10	10	*Streptomyces sp. CB00072*	WP_073867015.1
321	ksb_TLN-05220-Ile_13	13	*Micromonospora echinospora subsp. challisensis*	ADB23392.1
322	ksb_Trioxacarcin-Ile_10	10	*Streptomyces bottropensis*	AKT74261.1
323	ksb_Turbinmicin_13	13	*Micromonospora sp. WMMC415*	WP_155333335.1
324	ksb_Urdamycin_10	10	*Streptomyces fradiae*	CAA60570.1

325	ksb_UT-X26_F129_10	10	*Streptomyces aureus*	AYU66234.1
326	ksb_Warkmycin_10	10	*Streptomyces sp. CS057*	OWA25244.1
327	ksb_Wexrubicin_X	10	*Eubacteriales*	WP_025578805.1
328	ksb_WhiE_12	12	*Streptomyces coelicolor*	CAA39409.1
329	ksb_WS79089D_13	13	*Streptosporangium roseum DSM 43021*	WP_2890797.1
330	ksb_X26_10	10	*uncultured bacterium*	AEM44308.1
331	ksb_Xantholipin_13	13	*Streptomyces flavogriseus*	ADE22314.1
332	ksb_ZHMpentangular_13	13	*Streptomyces viridochromogenes*	WP_003993029.1

Table S2. Index of sequences from Cluster A.

Cluster A_Product name	ClusterA_Index
FABF	0
ksa_Abx_13	2
ksa_Anthrabenzoxocinone_13	10
ksa_Calixanthomycin_12	24
ksa_Dutomycin_10	47
ksa_Huanglongmycin_9unknown	78
ksa_LandomycinE'_10unknown	98
ksa_LandomycinE_10unknown	99
ksa_Murayaquinone_10unknown	114
ksa_Oxytetracycline'_10unknown	122
ksa_R1128-Leu_8unknown	132
ksa_Rubrolone-Bu_8unknown	138
ksa_SaquayamycinA_10	142
ksa_Sch47554_10unknown	145
ksa_Simocyclinone'_10unknown	148
ksb_Piloquinone-Leu_9	293
ksb_Pradimicin_12	295
ksb_Rubrolone-Bu_8	304

Table S3. Clustering Information.

**Cluster**	**Number of sequences**	**PKS Sequence index**
KS_3	8	0, 24, 47, 78, 114, 132, 142, 145
KS_2	146	1, 3, 4, 5, 6, 7, 8, 9, 12-23, 25-33, 36-43, 45-46, 48-97, 100-109, 111-113, 115-121, 123-131, 133-137, 139-141, 143-144, 146-147, 149-160, 162-166
KS_0	7	2, 10, 98, 99, 122, 138, 148
KS_4	6	11, 34, 35, 44, 110, 161
CLF_5	53	167-169, 174-176, 178, 186, 195, 199, 202, 205, 211-212, 214, 219-220, 226, 229, 233-235, 237-238, 241, 243, 245, 248-249, 271-272, 277, 279, 281, 283, 288-290, 297-298, 302-303, 305-306, 310, 313, 317, 319, 325, 328, 330-332
CLF_1	99	170-174, 179-198, 203-204, 206-208, 213, 215-218, 221-225, 228, 230-232, 236, 239-240, 242, 244, 246-247, 250-270, 273-274, 278, 280, 282, 284-287, 291-292, 294, 299-301, 307-309, 311-312, 314-315, 318, 320-324, 326, 329
CLF_4	6	177, 200, 201, 210, 276, 327
CLF_3	4	209, 227, 275, 316
CLF_0	3	293, 295, 304
CLF_2	1	296

Table S4. Edge weights between clusters.

Edges	Weights
CLF0 to KS0	1638452.91
KS0 to KS3	105408.77
CLF0 to KS2	5634.28
CLF0 to CLF5	350.86
KS3 to KS3	131.33
CLF0 to CLF3	128.58
CLF4 to KS4	109.02
KS3 to CLF4	58.28
KS4 to KS5	44.23
CLF5 to CLF1	41.93
CLF4 to KS2	26.98
KS4 to KS2	21.07
KS3 to KS2	18.38
CLF0 to KS3	17.22
CLF2 to KS2	14.00
CLF4 to KS1	10.36
KS3 to KS4	8.38
CLF4 to CLF2	4.27
KS0 to CLF3	2.31
KS3 to CLF1	2.00
CLF4 to CLF5	1.00
CLF1 to KS2	1.00

Table S5. Index of Non-oxidative PKS sequences.

Non-oxidative PKS product	KS_index	CLF_index
AQ-256_8	11	177
Clostrubin'_X	34	200
Clostrubin_X	35	201
Dendrubin_9	44	210
Pyxidicycline_9	130	296
Resistomycin_X	134	300
Wexrubicin_X	161	327

Table S9. Co-evolution distances to ancestral node, the ancestral node position was determined by averaging all vectors in Cluster A.

KS / CLF pair	KS to Cluster A distance	CLF to Cluster A distance	KS / CLF distance ratio
KS 1 / CLF 167	0.18	0.17	1.09
KS 3 / CLF 169	0.18	0.18	1.01
KS 4 / CLF 170	0.17	0.19	0.89
KS 5 / CLF 171	0.18	0.18	0.99
KS 6 / CLF 172	0.18	0.18	1.00
KS 7 / CLF 173	0.19	0.18	1.03
KS 8 / CLF 174	0.18	0.18	1.04
KS 9 / CLF 175	0.18	0.18	1.00
KS 11 / CLF 177	0.21	0.22	0.95
KS 12 / CLF 178	0.18	0.18	0.98
KS 13 / CLF 179	0.18	0.18	1.02
KS 14 / CLF 180	0.18	0.18	1.02
KS 15 / CLF 181	0.18	0.18	0.99
KS 16 / CLF 182	0.18	0.18	1.00
KS 17 / CLF 183	0.18	0.19	0.96
KS 18 / CLF 184	0.18	0.18	1.01
KS 19 / CLF 185	0.18	0.18	1.01
KS 20 / CLF 186	0.18	0.18	1.00
KS 21 / CLF 187	0.18	0.16	1.12
KS 22 / CLF 188	0.18	0.18	1.02
KS 23 / CLF 189	0.18	0.19	0.96
KS 25 / CLF 191	0.18	0.19	0.96
KS 26 / CLF 192	0.18	0.18	0.96
KS 27 / CLF 193	0.17	0.18	0.95
KS 28 / CLF 194	0.18	0.18	0.96
KS 29 / CLF 195	0.18	0.18	1.02
KS 30 / CLF 196	0.18	0.19	0.95
KS 31 / CLF 197	0.18	0.18	1.00
KS 32 / CLF 198	0.18	0.18	1.00
KS 33 / CLF 199	0.18	0.18	0.99
KS 34 / CLF 200	0.22	0.22	1.01
KS 35 / CLF 201	0.22	0.21	1.02
KS 36 / CLF 202	0.18	0.18	0.98
KS 37 / CLF 203	0.18	0.18	0.98
KS 38 / CLF 204	0.18	0.18	1.00
KS 39 / CLF 205	0.18	0.18	0.97
KS 40 / CLF 206	0.18	0.18	1.00
KS 41 / CLF 207	0.18	0.18	0.98

KS 42 / CLF 208	0.18	0.18	0.96
KS 43 / CLF 209	0.18	0.13	1.38
KS 44 / CLF 210	0.21	0.2	1.02
KS 45 / CLF 211	0.18	0.18	1.03
KS 46 / CLF 212	0.18	0.18	1.03
KS 48 / CLF 214	0.18	0.18	1.02
KS 49 / CLF 215	0.18	0.18	1.01
KS 50 / CLF 216	0.18	0.18	0.99
KS 51 / CLF 217	0.18	0.17	1.05
KS 52 / CLF 218	0.18	0.18	1.02
KS 53 / CLF 219	0.18	0.18	1.00
KS 54 / CLF 220	0.18	0.19	0.97
KS 55 / CLF 221	0.18	0.19	0.95
KS 56 / CLF 222	0.18	0.18	0.96
KS 57 / CLF 223	0.18	0.17	1.02
KS 58 / CLF 224	0.18	0.17	1.03
KS 59 / CLF 225	0.18	0.18	0.98
KS 60 / CLF 226	0.18	0.18	1.03
KS 61 / CLF 227	0.18	0.15	1.24
KS 62 / CLF 228	0.18	0.18	0.99
KS 63 / CLF 229	0.18	0.18	0.97
KS 64 / CLF 230	0.18	0.18	0.98
KS 65 / CLF 231	0.18	0.19	0.98
KS 66 / CLF 232	0.18	0.18	0.99
KS 67 / CLF 233	0.18	0.18	0.99
KS 68 / CLF 234	0.18	0.17	1.04
KS 69 / CLF 235	0.18	0.18	1.03
KS 70 / CLF 236	0.18	0.18	1.00
KS 71 / CLF 237	0.18	0.18	1.01
KS 72 / CLF 238	0.18	0.18	1.00
KS 73 / CLF 239	0.18	0.18	0.96
KS 74 / CLF 240	0.18	0.18	0.95
KS 75 / CLF 241	0.18	0.18	1.01
KS 76 / CLF 242	0.18	0.18	1.01
KS 77 / CLF 243	0.19	0.18	1.04
KS 79 / CLF 245	0.18	0.18	1.00
KS 80 / CLF 246	0.18	0.19	0.98
KS 81 / CLF 247	0.18	0.18	1.02
KS 82 / CLF 248	0.18	0.18	0.98
KS 83 / CLF 249	0.18	0.18	0.96
KS 84 / CLF 250	0.18	0.18	0.98
KS 85 / CLF 251	0.18	0.18	1.03
KS 86 / CLF 252	0.18	0.18	1.00

KS 87 / CLF 253	0.18	0.18	1.00
KS 88 / CLF 254	0.18	0.18	1.01
KS 89 / CLF 255	0.18	0.18	0.97
KS 90 / CLF 256	0.18	0.19	0.98
KS 91 / CLF 257	0.18	0.18	0.99
KS 92 / CLF 258	0.18	0.19	0.96
KS 93 / CLF 259	0.18	0.19	0.96
KS 94 / CLF 260	0.18	0.19	0.97
KS 95 / CLF 261	0.18	0.18	1.00
KS 96 / CLF 262	0.19	0.18	1.02
KS 97 / CLF 263	0.18	0.18	0.99
KS 100 / CLF 266	0.17	0.18	0.98
KS 101 / CLF 267	0.18	0.19	0.96
KS 102 / CLF 268	0.18	0.19	0.98
KS 103 / CLF 269	0.18	0.19	0.97
KS 104 / CLF 270	0.18	0.18	0.97
KS 105 / CLF 271	0.18	0.18	0.97
KS 106 / CLF 272	0.18	0.18	1.00
KS 107 / CLF 273	0.18	0.19	0.97
KS 108 / CLF 274	0.18	0.18	0.98
KS 109 / CLF 275	0.18	0.13	1.44
KS 110 / CLF 276	0.2	0.19	1.04
KS 111 / CLF 277	0.19	0.18	1.02
KS 112 / CLF 278	0.18	0.18	0.99
KS 113 / CLF 279	0.18	0.18	0.99
KS 115 / CLF 281	0.18	0.18	1.05
KS 116 / CLF 282	0.18	0.19	0.98
KS 117 / CLF 283	0.18	0.18	0.99
KS 118 / CLF 284	0.18	0.18	0.99
KS 119 / CLF 285	0.18	0.18	1.02
KS 120 / CLF 286	0.18	0.19	0.98
KS 121 / CLF 287	0.19	0.19	1.00
KS 123 / CLF 289	0.18	0.18	1.01
KS 124 / CLF 290	0.18	0.17	1.04
KS 125 / CLF 291	0.18	0.18	0.98
KS 126 / CLF 292	0.18	0.18	1.03
KS 127 / CLF 293	0.18	0.23	0.79
KS 128 / CLF 294	0.18	0.18	0.99
KS 129 / CLF 295	0.18	0.11	1.65
KS 130 / CLF 296	0.19	0.18	1.04
KS 131 / CLF 297	0.18	0.18	1.03
KS 133 / CLF 299	0.18	0.18	1.01
KS 134 / CLF 300	0.19	0.18	1.06

KS 135 / CLF 301	0.18	0.18	1.01
KS 136 / CLF 302	0.18	0.19	0.96
KS 137 / CLF 303	0.18	0.18	0.97
KS 139 / CLF 305	0.18	0.18	0.97
KS 140 / CLF 306	0.18	0.18	0.98
KS 141 / CLF 307	0.18	0.18	0.99
KS 143 / CLF 309	0.18	0.18	0.99
KS 144 / CLF 310	0.18	0.18	1.01
KS 146 / CLF 312	0.18	0.19	0.98
KS 147 / CLF 313	0.18	0.18	1.00
KS 149 / CLF 315	0.18	0.19	0.97
KS 150 / CLF 316	0.18	0.13	1.38
KS 151 / CLF 317	0.18	0.18	0.95
KS 152 / CLF 318	0.18	0.18	0.99
KS 153 / CLF 319	0.18	0.18	1.02
KS 154 / CLF 320	0.18	0.18	0.98
KS 155 / CLF 321	0.18	0.18	1.01
KS 156 / CLF 322	0.18	0.18	0.96
KS 157 / CLF 323	0.18	0.18	0.98
KS 158 / CLF 324	0.18	0.18	0.99
KS 159 / CLF 325	0.18	0.18	1.03
KS 160 / CLF 326	0.18	0.18	0.98
KS 161 / CLF 327	0.22	0.21	1.01
KS 162 / CLF 328	0.18	0.18	0.97
KS 163 / CLF 329	0.18	0.18	1.01
KS 164 / CLF 330	0.19	0.18	1.04
KS 165 / CLF 331	0.18	0.18	0.99
KS 166 / CLF 332	0.18	0.18	0.97

2. Supplementary Figures

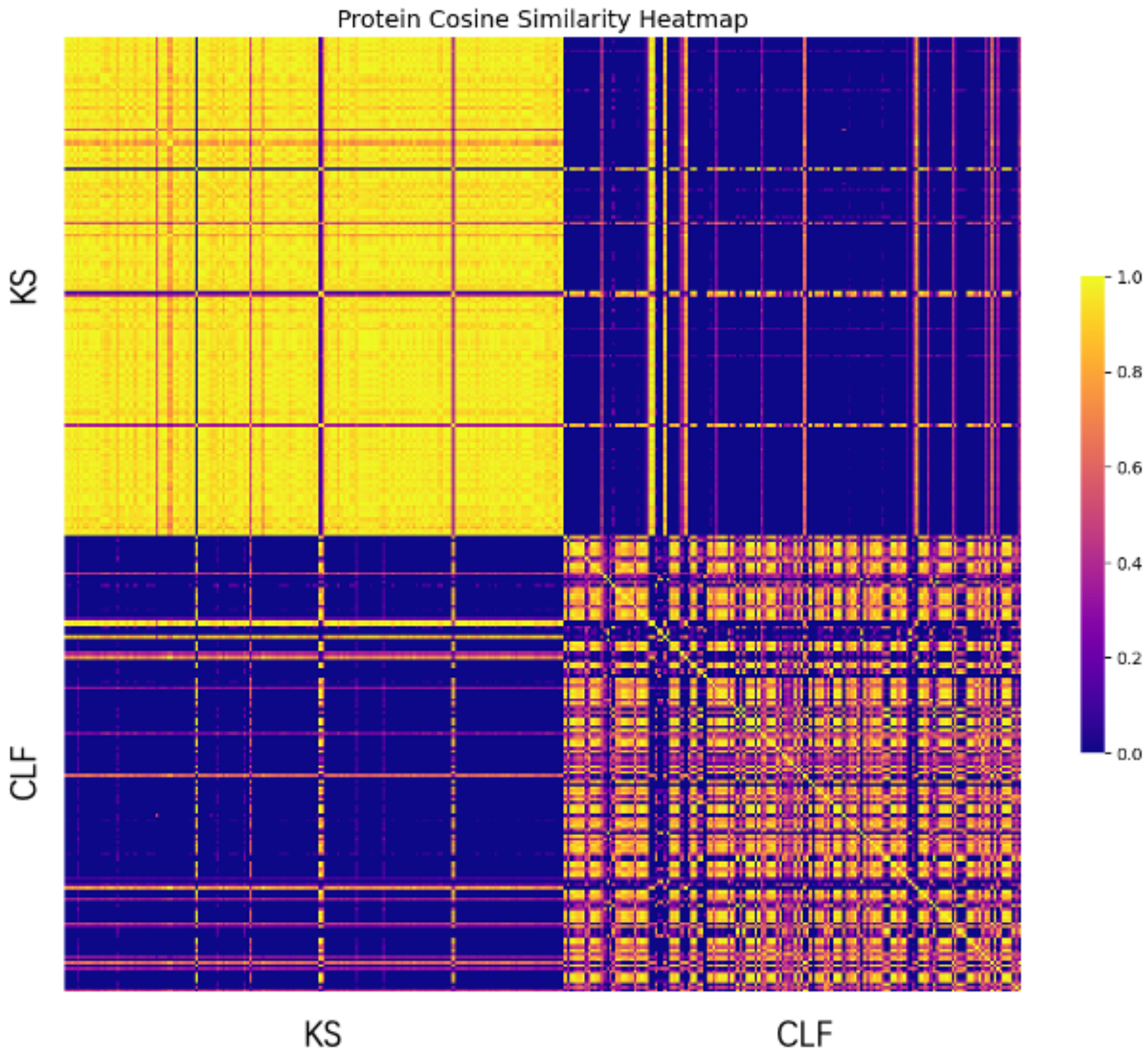

Fig. S1 Visualization of DeepFRI predicted function cosine similarities from all 166 pair of T2 PKS.

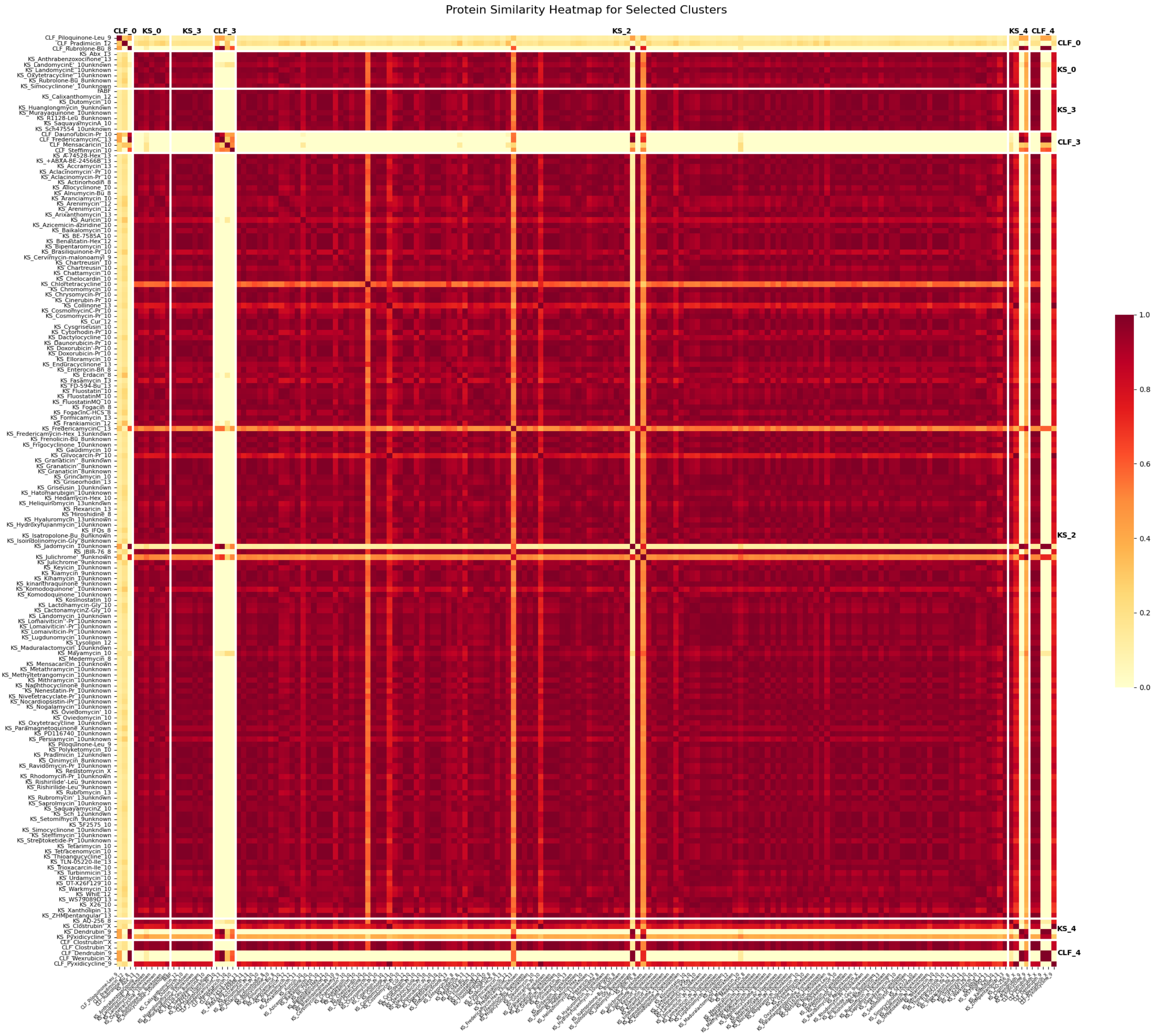

Fig. S2 Visualization of DeepFRI predicted function cosine similarities between selected clusters: CLF_0, KS_0, KS_3, CLF_3, KS_2, KS_4, CLF_4. KS_2 is the majority KS cluster.

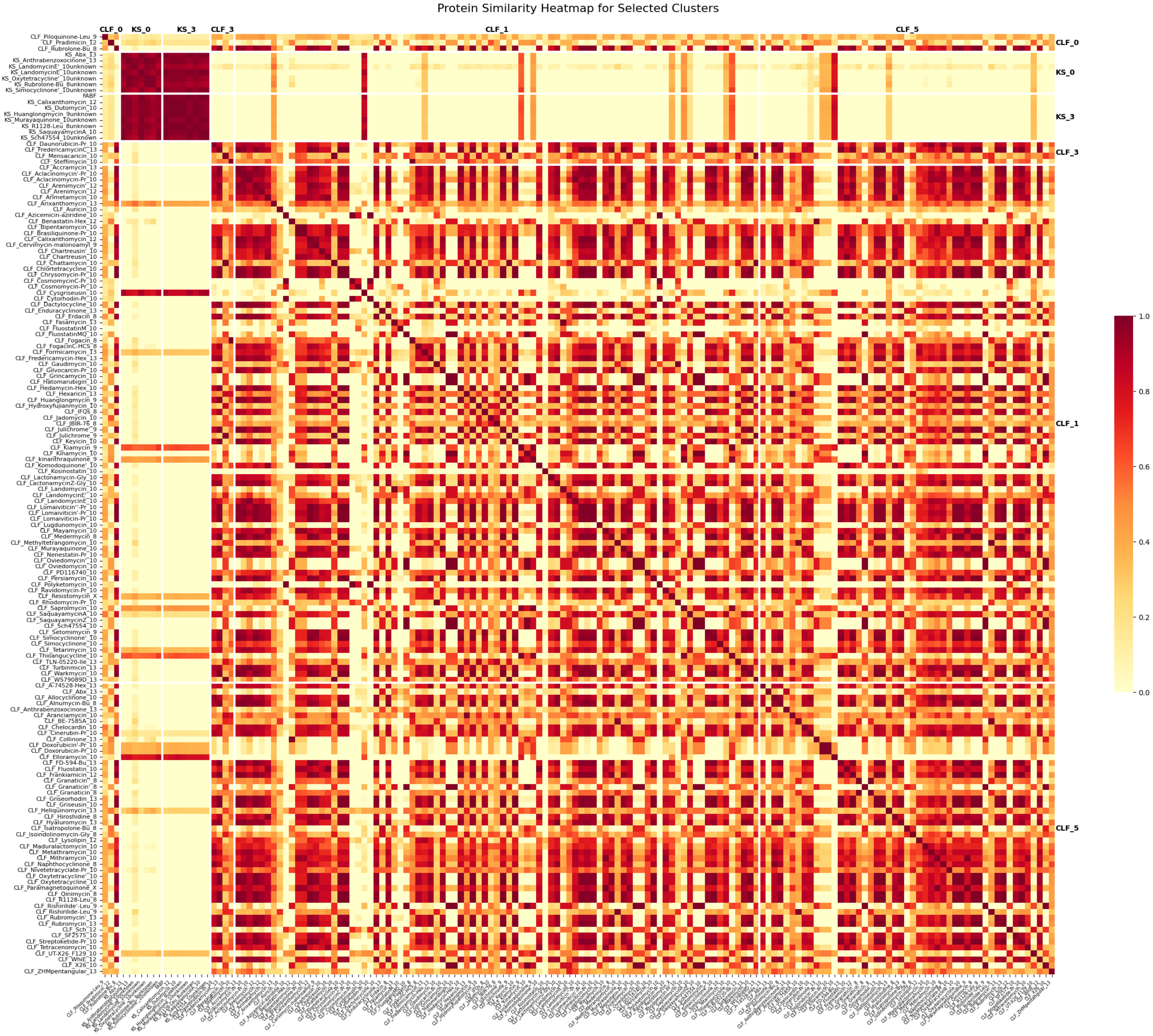

Fig. S3 Visualization of DeepFRI predicted function cosine similarities between selected clusters: CLF_0, KS_0, KS_3, CLF_3, CLF_1, CLF_5. CLF_1 and CLF_5 are the majority CLF clusters.
